# Gelatine as a Superior Hemostatic

**Published:** 1898-03

**Authors:** 


### GELATINE AS A SUPERIOR HEMOSTATIC.

An interesting and valuable discovery is the blood-coagulating quality of gelatine. This was made by Dastre and Floresco, as announced in the *Archives de Physiologie* of April, 1896, after the report of their experiments the preceding February before the Society of Biology. More recently, in the September 18th number of the *Presse medicale*, Dr. Paul Carnot takes up this subject, and in its consideration divides hemostatics in those which act by causing vascular contraction (like ergot) and those which seal the vessel openings by coagulating the blood (as styptics). He very properly objects to the use of the former whenever they can be avoided, for the reason that the plug of coagulated blood fitting the constricted vessel becomes too small after vascular dilatation, and thus leads to renewed bleeding.

That the gelatine solution causes actual coagulation of blood, and does not seal the vessels by merely gelatinizing, is proven by its being efficient in solutions too weak to permit gelatinizing. It is made up preferably with a sterilized normal salt solution, and to this an antiseptic may be added. Thus 7 parts of sodium chloride are added to 1000 parts of water. Gelatine is added to make a 5 to 10 per cent. solution. This is then boiled twice for fifteen minutes two days apart, care being observed not to let the temperature reach 239° F., as that temperature sometimes destroys its value.

He used this preparation in persistent nose-bleed in a child that had nearly bled itself out, and in which the usual styptics, such as the perchloride of iron, had proven unavailing. An injection of a 5-per-cent. gelatine solution stopped the bleeding at once. On the next day the other nostril bled, and it was stopped with equal promptness by the same means. This was effective despite the fact that the child had successive purpuric hemorrhages under the skin, and mucous and serous membranes, and finally died with only 365,000 red corpuscles to the cubic millimeter of blood.

The solution should be used at the bodily temperature, because if used hot it causes vascular contraction, thereby temporarily arresting the flow and presenting coagulation, or causing the formation of the small plugs in the contracted vessels that might lead to subsequent hemorrhage when the vessels again expanded, as in the case of those hemostatics acting by vascular contraction. It need not be given in hematemesis, because its blood-coagulating quality is destroyed by the gastic juice.

He has only used it once to arrest uterine hemorrhage, and that in a case of metrorrhagia caused by a fibroma. In this instance its action was wholly satisfactory. One point not to be forgotten is, that it must be injected into the cavity of the uterus to arrest hemorrhage from its walls, for it must come in direct contact with the blood as it leaves the vessels.

On experiments upon dogs, he repeatedly arrested bleeding from the freshly cut surface of the liver by a few seconds contact with the gelatine solution, but without exerting any pressure.

This is an agent that should not be overlooked in case of persistent bleeding. It is especially worthy of trial in severe uterine hemorrhage, especially post-partum. It is inexpensive, readily prepared, may be sterilized, and promises more than any other agent we have. But the caution to use it only at about the temperature of the body must not be overlooked. There are cases of menorrhagia and metrorrhagia in which it should be of great service, especially in such as are due to conditions only relievable by operation, and in which operation is refused, is postponed, or is no longer justifiable. By its means much time may be saved in perineal and cervical plastic operations in the prompt arrest of oozing, thus permitting the earlier approximation of the surfaces.

This new hemostatic is worthy of prompt and thorough trial, with the early recording of results.—*Medical Council.*

